# Best Practices for Scientific Computing

**DOI:** 10.1371/journal.pbio.1001745

**Published:** 2014-01-07

**Authors:** Greg Wilson, D. A. Aruliah, C. Titus Brown, Neil P. Chue Hong, Matt Davis, Richard T. Guy, Steven H. D. Haddock, Kathryn D. Huff, Ian M. Mitchell, Mark D. Plumbley, Ben Waugh, Ethan P. White, Paul Wilson

**Affiliations:** 1 Mozilla Foundation, Toronto, Ontario, Canada; 2 University of Ontario Institute of Technology, Oshawa, Ontario, Canada; 3 Michigan State University, East Lansing, Michigan, United States of America; 4 Software Sustainability Institute, Edinburgh, United Kingdom; 5 Space Telescope Science Institute, Baltimore, Maryland, United States of America; 6 University of Toronto, Toronto, Ontario, Canada; 7 Monterey Bay Aquarium Research Institute, Moss Landing, California, United States of America; 8 University of California Berkeley, Berkeley, California, United States of America; 9 University of British Columbia, Vancouver, British Columbia, Canada; 10 Queen Mary University of London, London, United Kingdom; 11 University College London, London, United Kingdom; 12 Utah State University, Logan, Utah, United States of America; 13 University of Wisconsin, Madison, Wisconsin, United States of America; University of California Davis, United States of America

## Abstract

We describe a set of best practices for scientific software development, based on research and experience, that will improve scientists' productivity and the reliability of their software.

## Introduction

Scientists spend an increasing amount of time building and using software. However, most scientists are never taught how to do this efficiently. As a result, many are unaware of tools and practices that would allow them to write more reliable and maintainable code with less effort. We describe a set of best practices for scientific software development that have solid foundations in research and experience, and that improve scientists' productivity and the reliability of their software.

Software is as important to modern scientific research as telescopes and test tubes. From groups that work exclusively on computational problems, to traditional laboratory and field scientists, more and more of the daily operation of science revolves around developing new algorithms, managing and analyzing the large amounts of data that are generated in single research projects, combining disparate datasets to assess synthetic problems, and other computational tasks.

Scientists typically develop their own software for these purposes because doing so requires substantial domain-specific knowledge. As a result, recent studies have found that scientists typically spend 30% or more of their time developing software [1],[2]. However, 90% or more of them are primarily self-taught [1],[2], and therefore lack exposure to basic software development practices such as writing maintainable code, using version control and issue trackers, code reviews, unit testing, and task automation.

We believe that software is just another kind of experimental apparatus [3] and should be built, checked, and used as carefully as any physical apparatus. However, while most scientists are careful to validate their laboratory and field equipment, most do not know how reliable their software is [4],[5]. This can lead to serious errors impacting the central conclusions of published research [6]: recent high-profile retractions, technical comments, and corrections because of errors in computational methods include papers in *Science* [7],[8], *PNAS* [9], the *Journal of Molecular Biology* [10], *Ecology Letters* [11],[12], the *Journal of Mammalogy* [13], *Journal of the American College of Cardiology* [14], *Hypertension* [15], and *The American Economic Review* [16].

In addition, because software is often used for more than a single project, and is often reused by other scientists, computing errors can have disproportionate impacts on the scientific process. This type of cascading impact caused several prominent retractions when an error from another group's code was not discovered until after publication [6]. As with bench experiments, not everything must be done to the most exacting standards; however, scientists need to be aware of best practices both to improve their own approaches and for reviewing computational work by others.

This paper describes a set of practices that are easy to adopt and have proven effective in many research settings. Our recommendations are based on several decades of collective experience both building scientific software and teaching computing to scientists [17],[18], reports from many other groups [19]–, guidelines for commercial and open source software development [26],, and on empirical studies of scientific computing [28]–[31] and software development in general (summarized in [32]). None of these practices will guarantee efficient, error-free software development, but used in concert they will reduce the number of errors in scientific software, make it easier to reuse, and save the authors of the software time and effort that can used for focusing on the underlying scientific questions.

Our practices are summarized in Box 1; labels in the main text such as “(1a)” refer to items in that summary. For reasons of space, we do not discuss the equally important (but independent) issues of reproducible research, publication and citation of code and data, and open science. We do believe, however, that all of these will be much easier to implement if scientists have the skills we describe.

**Box 1. Summary of Best Practices**

1. Write programs for people, not computers.
  - A program should not require its readers to hold more than a handful of facts in memory at once.
  - Make names consistent, distinctive, and meaningful.
  - Make code style and formatting consistent.
2. Let the computer do the work.
  - Make the computer repeat tasks.
  - Save recent commands in a file for re-use.
  - Use a build tool to automate workflows.
3. Make incremental changes.
  - Work in small steps with frequent feedback and course correction.
  - Use a version control system.
  - Put everything that has been created manually in version control.
4. Don't repeat yourself (or others).
  - Every piece of data must have a single authoritative representation in the system.
  - Modularize code rather than copying and pasting.
  - Re-use code instead of rewriting it.
5. Plan for mistakes.
  - Add assertions to programs to check their operation.
  - Use an off-the-shelf unit testing library.
  - Turn bugs into test cases.
  - Use a symbolic debugger.
6. Optimize software only after it works correctly.
  - Use a profiler to identify bottlenecks.
  - Write code in the highest-level language possible.
7. Document design and purpose, not mechanics.
  - Document interfaces and reasons, not implementations.
  - Refactor code in preference to explaining how it works.
  - Embed the documentation for a piece of software in that software.
8. Collaborate.
  - Use pre-merge code reviews.
  - Use pair programming when bringing someone new up to speed and when tackling particularly tricky problems.
  - Use an issue tracking tool.

## Write Programs for People, Not Computers

Scientists writing software need to write code that both executes correctly and can be easily read and understood by other programmers (especially the author's future self). If software cannot be easily read and understood, it is much more difficult to know that it is actually doing what it is intended to do. To be productive, software developers must therefore take several aspects of human cognition into account: in particular, that human working memory is limited, human pattern matching abilities are finely tuned, and human attention span is short [33]–[37].

First, ***a program should not require its readers to hold more than a handful of facts in memory at once (1a)***. Human working memory can hold only a handful of items at a time, where each item is either a single fact or a “chunk” aggregating several facts [33],[34], so programs should limit the total number of items to be remembered to accomplish a task. The primary way to accomplish this is to break programs up into easily understood functions, each of which conducts a single, easily understood, task. This serves to make each piece of the program easier to understand in the same way that breaking up a scientific paper using sections and paragraphs makes it easier to read.

Second, scientists should ***make names consistent, distinctive, and meaningful (1b)***. For example, using non-descriptive names, like a and foo, or names that are very similar, like results and results2, is likely to cause confusion.

Third, scientists should ***make code style and formatting consistent (1c)***. If different parts of a scientific paper used different formatting and capitalization, it would make that paper more difficult to read. Likewise, if different parts of a program are indented differently, or if programmers mix CamelCaseNaming and pothole_case_naming, code takes longer to read and readers make more mistakes [35],[36].

## Let the Computer Do the Work

Science often involves repetition of computational tasks such as processing large numbers of data files in the same way or regenerating figures each time new data are added to an existing analysis. Computers were invented to do these kinds of repetitive tasks but, even today, many scientists type the same commands in over and over again or click the same buttons repeatedly [17]. In addition to wasting time, sooner or later even the most careful researcher will lose focus while doing this and make mistakes.

Scientists should therefore ***make the computer repeat tasks (2a)*** and ***save recent commands in a file for re-use (2b)***. For example, most command-line tools have a “history” option that lets users display and re-execute recent commands, with minor edits to filenames or parameters. This is often cited as one reason command-line interfaces remain popular [38],[39]: “do this again” saves time and reduces errors.

A file containing commands for an interactive system is often called a *script*, though there is real no difference between this and a program. When these scripts are repeatedly used in the same way, or in combination, a workflow management tool can be used. The paradigmatic example is compiling and linking programs in languages such as Fortran, C++, Java, and C# [40]. The most widely used tool for this task is probably Make (http://www.gnu.org/software/make), although many alternatives are now available [41]. All of these allow people to express dependencies between files, i.e., to say that if A or B has changed, then C needs to be updated using a specific set of commands. These tools have been successfully adopted for scientific workflows as well [42].

To avoid errors and inefficiencies from repeating commands manually, we recommend that scientists ***use a build tool to automate workflows (2c)***, e.g., specify the ways in which intermediate data files and final results depend on each other, and on the programs that create them, so that a single command will regenerate anything that needs to be regenerated.

In order to maximize reproducibility, everything needed to re-create the output should be recorded automatically in a format that other programs can read. (Borrowing a term from archaeology and forensics, this is often called the *provenance* of data.) There have been some initiatives to automate the collection of this information, and standardize its format [43], but it is already possible to record the following without additional tools:

- unique identifiers and version numbers for raw data records (which scientists may need to create themselves);
- unique identifiers and version numbers for programs and libraries;
- the values of parameters used to generate any given output; and
- the names and version numbers of programs (however small) used to generate those outputs.

## Make Incremental Changes

Unlike traditional commercial software developers, but very much like developers in open source projects or startups, scientific programmers usually don't get their requirements from customers, and their requirements are rarely frozen [31],[44]. In fact, scientists often *can't* know what their programs should do next until the current version has produced some results. This challenges design approaches that rely on specifying requirements in advance.

Programmers are most productive when they ***work in small steps with frequent feedback and course correction (3a)*** rather than trying to plan months or years of work in advance. While the details vary from team to team, these developers typically work in steps that are sized to be about an hour long, and these steps are often grouped in iterations that last roughly one week. This accommodates the cognitive constraints discussed in the first section, and acknowledges the reality that real-world requirements are constantly changing. The goal is to produce working (if incomplete) code after each iteration. While these practices have been around for decades, they gained prominence starting in the late 1990s under the banner of *agile development* [45],[46].

Two of the biggest challenges scientists and other programmers face when working with code and data are keeping track of changes (and being able to revert them if things go wrong), and collaborating on a program or dataset [23]. Typical solutions are to email software to colleagues or to copy successive versions of it to a shared folder, e.g., Dropbox (http://www.dropbox.com). However, both approaches are fragile and can lead to confusion and lost work when important changes are overwritten or out-of-date files are used. It's also difficult to find out which changes are in which versions or to say exactly how particular results were computed at a later date.

The standard solution in both industry and open source is to ***use a version control system (3b)*** (VCS) [27],[47]. A VCS stores snapshots of a project's files in a *repository* (or a set of repositories). Programmers can modify their working copy of the project at will, then *commit* changes to the repository when they are satisfied with the results to share them with colleagues.

Crucially, if several people have edited files simultaneously, the VCS highlights the differences and requires them to resolve any conflicts before accepting the changes. The VCS also stores the entire history of those files, allowing arbitrary versions to be retrieved and compared, together with metadata such as comments on what was changed and the author of the changes. All of this information can be extracted to provide provenance for both code and data.

Many good VCSes are open source and freely available, including Git (http://git-scm.com), Subversion (http://subversion.apache.org), and Mercurial (http://mercurial.selenic.com). Many free hosting services are available as well, with GitHub (https://github.com), BitBucket (https://bitbucket.org), SourceForge (http://sourceforge.net), and Google Code (http://code.google.com) being the most popular. As with coding style, the best one to use is almost always whatever your colleagues are already using [27].

Reproducibility is maximized when scientists ***put everything that has been created manually in version control (3c)***, including programs, original field observations, and the source files for papers. Automated output and intermediate files can be regenerated as needed. Binary files (e.g., images and audio clips) may be stored in version control, but it is often more sensible to use an archiving system for them, and store the metadata describing their contents in version control instead [48].

## Don't Repeat Yourself (or Others)

Anything that is repeated in two or more places is more difficult to maintain. Every time a change or correction is made, multiple locations must be updated, which increases the chance of errors and inconsistencies. To avoid this, programmers follow the DRY Principle [49], for “don't repeat yourself,” which applies to both data and code.

For data, this maxim holds that ***every piece of data must have a single authoritative representation in the system (4a)***. Physical constants ought to be defined exactly once to ensure that the entire program is using the same value; raw data files should have a single canonical version, every geographic location from which data has been collected should be given an ID that can be used to look up its latitude and longitude, and so on.

The DRY Principle applies to code at two scales. At small scales, ***modularize code rather than copying and pasting (4b)***. Avoiding “code clones” has been shown to reduce error rates [50]: when a change is made or a bug is fixed, that change or fix takes effect everywhere, and people's mental model of the program (i.e., their belief that “this one's been fixed”) remains accurate. As a side effect, modularizing code allows people to remember its functionality as a single mental chunk, which in turn makes code easier to understand. Modularized code can also be more easily repurposed for other projects.

At larger scales, it is vital that scientific programmers ***re-use code instead of rewriting it (4c)***. Tens of millions of lines of high-quality open source software are freely available on the web, and at least as much is available commercially. It is typically better to find an established library or package that solves a problem than to attempt to write one's own routines for well established problems (e.g., numerical integration, matrix inversions, etc.).

## Plan for Mistakes

Mistakes are inevitable, so verifying and maintaining the validity of code over time is immensely challenging [51]. While no single practice has been shown to catch or prevent all mistakes, several are very effective when used in combination [47],[52],[53].

The first line of defense is *defensive programming*. Experienced programmers ***add assertions to programs to check their operation (5a)*** because experience has taught them that everyone (including their future self) makes mistakes. An *assertion* is simply a statement that something holds true at a particular point in a program; as the example below shows, assertions can be used to ensure that inputs are valid, outputs are consistent, and so on.
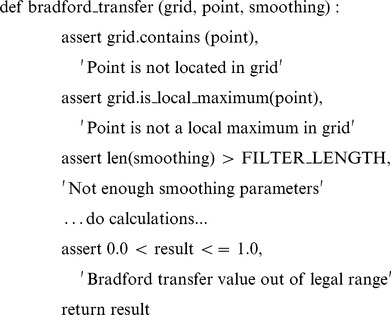
Assertions can make up a sizeable fraction of the code in well-written applications, just as tools for calibrating scientific instruments can make up a sizeable fraction of the equipment in a lab. These assertions serve two purposes. First, they ensure that if something does go wrong, the program will halt immediately, which simplifies debugging. Second, assertions are *executable documentation*, i.e., they explain the program as well as checking its behavior. This makes them more useful in many cases than comments since the reader can be sure that they are accurate and up to date.

The second layer of defense is *automated testing*. Automated tests can check to make sure that a single unit of code is returning correct results (*unit tests*), that pieces of code work correctly when combined (*integration tests*), and that the behavior of a program doesn't change when the details are modified (*regression tests*). These tests are conducted by the computer, so that they are easy to rerun every time the program is modified. Creating and managing tests is easier if programmers ***use an off-the-shelf unit testing library (5b)*** to initialize inputs, run tests, and report their results in a uniform way. These libraries are available for all major programming languages including those commonly used in scientific computing [54]–[56].

Tests check to see whether the code matches the researcher's expectations of its behavior, which depends on the researcher's understanding of the problem at hand [57]–[59]. For example, in scientific computing, tests are often conducted by comparing output to simplified cases, experimental data, or the results of earlier programs that are trusted. Another approach for generating tests is to ***turn bugs into test cases (5c)*** by writing tests that trigger a bug that has been found in the code and (once fixed) will prevent the bug from reappearing unnoticed. In combination these kinds of testing can improve our confidence that scientific code is operating properly and that the results it produces are valid. An additional benefit of testing is that it encourages programmers to design and build code that is testable (i.e., self-contained functions and classes that can run more or less independently of one another). Code that is designed this way is also easier to understand and more reusable.

No matter how good one's computational practice is, reasonably complex code will always initially contain bugs. Fixing bugs that have been identified is often easier if you ***use a symbolic debugger (5d)*** to track them down. A better name for this kind of tool would be “interactive program inspector” since a debugger allows users to pause a program at any line (or when some condition is true), inspect the values of variables, and walk up and down active function calls to figure out why things are behaving the way they are. Debuggers are usually more productive than adding and removing print statements or scrolling through hundreds of lines of log output [60], because they allow the user to see exactly how the code is executing rather than just snapshots of state of the program at a few moments in time. In other words, the debugger allows the scientist to witness what is going wrong directly, rather than having to anticipate the error or infer the problem using indirect evidence.

## Optimize Software Only after It Works Correctly

Today's computers and software are so complex that even experts find it hard to predict which parts of any particular program will be performance bottlenecks [61]. The most productive way to make code fast is therefore to make it work correctly, determine whether it's actually worth speeding it up, and—in those cases where it is—to ***use a profiler to identify bottlenecks (6a)***.

This strategy also has interesting implications for choice of programming language. Research has confirmed that most programmers write roughly the same number of lines of code per unit time regardless of the language they use [62]. Since faster, lower level, languages require more lines of code to accomplish the same task, scientists are most productive when they ***write code in the highest-level language possible (6b)***, and shift to low-level languages like C and Fortran only when they are sure the performance boost is needed. (Using higher-level languages also helps program comprehensibility, since such languages have, in a sense, “pre-chunked” the facts that programmers need to have in short-term memory.)

Taking this approach allows more code to be written (and tested) in the same amount of time. Even when it is known before coding begins that a low-level language will ultimately be necessary, rapid prototyping in a high-level language helps programmers make and evaluate design decisions quickly. Programmers can also use a high-level prototype as a test oracle for a high-performance low-level reimplementation, i.e., compare the output of the optimized (and usually more complex) program against the output from its unoptimized (but usually simpler) predecessor in order to check its correctness.

## Document Design and Purpose, Not Mechanics

In the same way that a well documented experimental protocol makes research methods easier to reproduce, good documentation helps people understand code. This makes the code more reusable and lowers maintenance costs [47]. As a result, code that is well documented makes it easier to transition when the graduate students and postdocs who have been writing code in a lab transition to the next career phase. Reference documentation and descriptions of design decisions are key for improving the understandability of code. However, inline documentation that recapitulates code is *not* useful. Therefore we recommend that scientific programmers ***document interfaces and reasons, not implementations (7a)***. For example, a clear description like this at the beginning of a function that describes what it does and its inputs and outputs is useful:
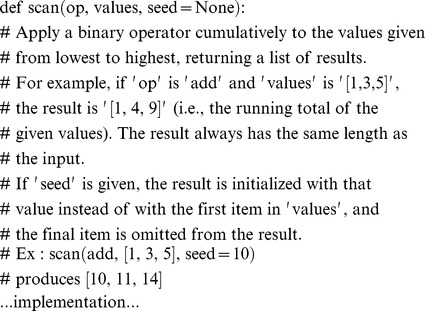
In contrast, the comment in the code fragment below does nothing to aid comprehension:
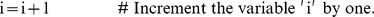
If a substantial description of the implementation of a piece of software is needed, it is better to ***refactor code in preference to explaining how it works (7b)***, i.e., rather than write a paragraph to explain a complex piece of code, reorganize the code itself so that it doesn't need such an explanation. This may not always be possible—some pieces of code are intrinsically difficult—but the onus should always be on the author to convince his or her peers of that.

The best way to create and maintain reference documentation is to ***embed the documentation for a piece of software in that software (7c)***. Doing this increases the probability that when programmers change the code, they will update the documentation at the same time.

Embedded documentation usually takes the form of specially-formatted and placed comments. Typically, a *documentation generator* such as Javadoc, Doxygen, or Sphinx extracts these comments and generates well-formatted web pages and other human-friendly documents (http://en.wikipedia.org/wiki/Comparison_of_documentation_generators). Alternatively, code can be embedded in a larger document that includes information about what the code is doing (i.e., literate programming). Common approaches to this include this use of knitr [63] and IPython Notebooks [64].

## Collaborate

In the same way that having manuscripts reviewed by other scientists can reduce errors and make research easier to understand, reviews of source code can eliminate bugs and improve readability. A large body of research has shown that *code reviews* are the most cost-effective way of finding bugs in code [65],[66]. They are also a good way to spread knowledge and good practices around a team. In projects with shifting membership, such as most academic labs, code reviews help ensure that critical knowledge isn't lost when a student or postdoc leaves the lab.

Code can be reviewed either before or after it has been committed to a shared version control repository. Experience shows that if reviews don't have to be done in order to get code into the repository, they will soon not be done at all [27]. We therefore recommend that projects ***use pre-merge code reviews (8a)***.

An extreme form of code review is *pair programming*, in which two developers sit together while writing code. One (the driver) actually writes the code; the other (the navigator) provides real-time feedback and is free to track larger issues of design and consistency. Several studies have found that pair programming improves productivity [67], but many programmers find it intrusive. We therefore recommend that teams ***use pair programming when bringing someone new up to speed and when tackling particularly tricky problems (8b)***.

Once a team grows beyond a certain size, it becomes difficult to keep track of what needs to be reviewed, or of who's doing what. Teams can avoid a lot of duplicated effort and dropped balls if they ***use an issue tracking tool (8c)*** to maintain a list of tasks to be performed and bugs to be fixed [68]. This helps avoid duplicated work and makes it easier for tasks to be transferred to different people. Free repository hosting services like GitHub include issue tracking tools, and many good standalone tools exist as well, such as Trac (http://trac.edgewall.org).

## Conclusion

We have outlined a series of recommended best practices for scientific computing based on extensive research, as well as our collective experience. These practices can be applied to individual work as readily as group work; separately and together, they improve the productivity of scientific programming and the reliability of the resulting code, and therefore the speed with which we produce results and our confidence in them. They are also, we believe, prerequisites for reproducible computational research: if software is not version controlled, readable, and tested, the chances of its authors (much less anyone else) being able to re-create results are remote.

Our 24 recommendations are a beginning, not an end. Individuals and groups who have incorporated them into their work will find links to more advanced practices at Software Carpentry (http://software-carpentry.org).

Research suggests that the time cost of implementing these kinds of tools and approaches in scientific computing is almost immediately offset by the gains in productivity of the programmers involved [17]. Even so, the recommendations described above may seem intimidating to implement. Fortunately, the different practices reinforce and support one another, so the effort required is less than the sum of adding each component separately. Nevertheless, we do not recommend that research groups attempt to implement all of these recommendations at once, but instead suggest that these tools be introduced incrementally over a period of time.

How to implement the recommended practices can be learned from many excellent tutorials available online or through workshops and classes organized by groups like Software Carpentry. This type of training has proven effective at driving adoption of these tools in scientific settings [17],[69].

For computing to achieve the level of rigor that is expected throughout other parts of science, it is necessary for scientists to begin to adopt the tools and approaches that are known to improve both the quality of software and the efficiency with which it is produced. To facilitate this adoption, universities and funding agencies need to support the training of scientists in the use of these tools and the investment of time and money in building better scientific software. Investment in these approaches by both individuals and institutions will improve our confidence in the results of computational science and will allow us to make more rapid progress on important scientific questions than would otherwise be possible.

## References

[1] Hannay JE, Langtangen HP, MacLeod C, Pfahl D, Singer J, et al.. (2009) How do scientists develop and use scientific software? In: Proceedings Second International Workshop on Software Engineering for Computational Science and Engineering. pp. 1–8. doi:10.1109/SECSE.2009.5069155.

[2] Prabhu P, Jablin TB, Raman A, Zhang Y, Huang J, et al.. (2011) A survey of the practice of computational science. In: Proceedings 24th ACM/IEEE Conference on High Performance Computing, Networking, Storage and Analysis. pp. 19:1–19:12. doi:10.1145/2063348.2063374.

[3] Vardi M (2010) Science has only two legs. Communications of the ACM 53: 5.

[4] Hatton L, Roberts A (1994) How accurate is scientific software? IEEE T Software Eng 20: 785–797.

[5] Hatton L (1997) The T experiments: errors in scientific software. Computational Science & Engineering 4: 27–38.

[6] Merali Z (2010) Error: why scientific programming does not compute. Nature 467: 775–777.20944712

[7] Chang G, Roth CB, Reyes CL, Pornillos O, Chen YJ, et al (2006) Retraction. Science 314: 1875. 10.1126/science.314.5807.1875b 17185584

[8] Ferrari F, Jung YL, Kharchenko PV, Plachetka A, Alekseyenko AA, et al (2013) Comment on “Drosophila dosage compensation involves enhanced Pol II recruitment to male X-Linked promoters”. Science 340: 273. 10.1126/science.1231815 PMC3665607 23599463

[9] Ma C, Chang G (2007) Retraction for Ma and Chang, structure of the multidrug resistance efflux transporter EmrE from *Escherichia coli* . Proc Natl Acad Sci U S A 104: 3668. 10.1073/pnas.0700711104 PMC1805606 17360700

[10] Chang G (2007) Retraction of ‘Structure of MsbA from Vibrio cholera: A Multidrug Resistance ABC Transporter Homolog in a Closed Conformation’ [J. Mol. Biol. (2003) 330 419430]. Journal of Molecular Biology 369: 596. 17580380 10.1016/j.jmb.2003.05.001

[11] Lees DC, Colwell RK (2007) A strong Madagascan rainforest MDE and no equatorward increase in species richness: re-analysis of ‘The Missing Madagascan Mid-Domain Effect’, by Kerr JT, Perring M, Currie DJ (Ecol Lett 9:149159, 2006). Ecol Lett 10: E4–E8. 17663706 10.1111/j.1461-0248.2007.01040.x

[12] Currie D, Kerr J (2007) Testing, as opposed to supporting, the mid-domain hypothesis: a response to lees and colwell. Ecol Lett 10: E9–E10.

[13] Kelt DA, Wilson JA, Konno ES, Braswell JD, Deutschman D (2008) Differential responses of two species of kangaroo rat (Dipodomys) to heavy rains: a humbling reappraisal. J Mammal 89: 252–254.

[14] Anon (2013) Retraction notice to “Plasma PCSK9 levels and clinical outcomes in the TNT (Treating to New Targets) Trial” [J Am Coll Cardiol 2012;59:17781784]. J Am Coll Cardiol 61: 1751. 23729038 10.1016/j.jacc.2013.04.002

[15] Hypertension 60: e29. Retraction. Implications of new hypertension guidelines in the United States. Retraction of Bertoia ML, Waring ME, Gupta PS, Roberts MB, Eaton CB. Hypertension (2011) 58: 361–366. 10.1161/HYPERTENSIONAHA.111.175463 21768528

[16] Herndon T, Ash M, Pollin R (2013). Does high public debt consistently stifle economic growth? A critique of Reinhart and Rogoff. Working paper, Political Economy Research Institute. Available: http://www.peri.umass.edu/fileadmin/pdf/working papers/working papers 301-350/WP322.pdf.

[17] Aranda J (2012). Software carpentry assessment report. Available: http://software-carpentry.org/papers/arandaassessment-2012-07.pdf.

[18] Wilson G (2006) Software carpentry: getting scientists to write better code by making them more productive. Comput Sci Eng 66–69.

[19] Heroux MA, Willenbring JM (2009) Barely-sufficient software engineering: 10 practices to improve your CSE software. In: Proceedings Second International Workshop on Software Engineering for Computational Science and Engineering. pp. 15–21. 10.1109/SECSE.2009.5069157.

[20] Kane D (2003) Introducing agile development into bioinformatics: an experience report. In: Proceedings of the Conference on Agile Development, IEEE Computer Society, Washington (D.C.); 2003, 0-7695-2013-8, pp. 132–139, 10.1109/ADC.2003.1231463.

[21] Kane D, Hohman M, Cerami E, McCormick M, Kuhlmman K, et al (2006) Agile methods in biomedical software development: a multi-site experience report. BMC Bioinformatics 7: 273. 16734914 10.1186/1471-2105-7-273 PMC1539031

[22] Killcoyne S, Boyle J (2009) Managing chaos: lessons learned developing software in the life sciences. Comput Sci Eng 11: 20–29. 20700479 10.1109/MCSE.2009.198 PMC2917833

[23] Matthews D, Wilson G, Easterbrook S (2008) Configuration management for large-scale scientific computing at the UK Met office. Comput Sci Eng 56–64.

[24] Pitt-Francis J, Bernabeu MO, Cooper J, Garny A, Momtahan L, et al (2008) Chaste: using agile programming techniques to develop computational biology software. Philos Trans A Math Phys Eng Sci 366: 3111–3136. 18565813 10.1098/rsta.2008.0096

[25] Pouillon Y, Beuken JM, Deutsch T, Torrent M, Gonze X (2011) Organizing software growth and distributed development: the case of abinit. Comput Sci Eng 13: 62–69.

[26] Spolsky J (2000). The Joel test: 12 steps to better code. Available: http://www.joelonsoftware.com/articles/fog0000000043.html. Accessed September 2013.

[27] Fogel K (2005) Producing open source software: how to run a successful free software project. Sepastopol (California): O'Reilly. Available: http://producingoss.com.

[28] Carver JC, Kendall RP, Squires SE, Post DE (2007) Software development environments for scientific and engineering software: a series of case studies. In: Proceedings 29th International Conference on Software Engineering. pp. 550–559. 10.1109/ICSE.2007.77.

[29] Kelly D, Hook D, Sanders R (2009) Five recommended practices for computational scientists who write software. Comput Sci Eng 11: 48–53.

[30] Segal J (2005) When software engineers met research scientists: a case study. Empir Softw Eng 10: 517–536.

[31] Segal J (2008) Models of scientific software development. In: Proceedings First International Workshop on Software Engineering for Computational Science and Engineering. Available: http://secse08.cs.ua.edu/Papers/Segal.pdf.

[32] Oram A, Wilson G, editors(2010) Making software: what really works, and why we believe it. Sepastopol (California): O'Reilly.

[33] Baddeley A, Eysenck MW, Anderson MC (2009) Memory. New York: Psychology Press.

[34] Hock RR (2008) Forty studies that changed psychology: explorations into the history of psychological research, 6th edition. Upper Saddle River (New Jersey): Prentice Hall.

[35] Letovsky S (1986) Cognitive processes in program comprehension. In: Proceedings First Workshop on Empirical Studies of Programmers. pp. 58–79. 10.1016/0164-1212(87)90032-X.

[36] Binkley D, Davis M, Lawrie D, Morrell C (2009) To CamelCase or under_score. In: Proceedings 2009 IEEE International Conference on Program Comprehension. pp. 158–167. 10.1109/ICPC.2009.5090039.

[37] Robinson E (2005) Why crunch mode doesn't work: six lessons. Available: http://www.igda.org/why-crunch-modes-doesnt-work-six-lessons. Accessed: September 2013.

[38] Ray DS, Ray EJ (2009) Unix and Linux: visual quickstart guide. 4th edition. San Francisco: Peachpit Press.

[39] Haddock S, Dunn C (2010) Practical computing for biologists. Sunderland (Massachusetts): Sinauer Associates.

[40] Dubois PF, Epperly T, Kumfert G (2003) Why Johnny can't build (portable scientific software). Comput Sci Eng 5: 83–88.

[41] Smith P (2011) Software build systems: principles and experience. Boston: Addison-Wesley.

[42] Fomel S, Hennenfent G (2007) Reproducible computational experiments using SCons. In: Proceedings 32nd International Conference on Acoustics, Speech, and Signal Processing. volume IV, pp. 1257–1260. 10.1109/ICASSP.2007.367305.

[43] Moreau L, Freire J, Futrelle J, McGrath RE, Myers J, et al.. (2007) The open provenance model (v1.00). Technical report, University of Southampton. Accessed September 2013.

[44] Segal J, Morris C (2008) Developing scientific software. IEEE Software 25: 18–20.

[45] Martin RC (2002) Agile software development, principles, patterns, and practices. Upper Saddle River (New Jersey): Prentice Hall.

[46] Kniberg H (2007) Scrum and XP from the trenches. C4Media, 978-1-4303-2264-1, Available from http://www.infoq.com/minibooks/scrum-xp-from-the-trenches.

[47] McConnell S (2004) Code complete: a practical handbook of software construction, 2nd edition. Seattle: Microsoft Press.

[48] Noble WS (2009) A quick guide to organizing computational biology projects. PLoS Comput Biol 5: e1000424 doi:10.1371/journal.pcbi.1000424 19649301 10.1371/journal.pcbi.1000424 PMC2709440

[49] Hunt A, Thomas D (1999) The pragmatic programmer: from journeyman to master. Boston: Addison-Wesley.

[50] Juergens E, Deissenboeck F, Hummel B, Wagner S (2009) Do code clones matter? In: Proceedings 31st International Conference on Software Engineering. pp. 485–495. 10.1109/ICSE.2009.5070547.

[51] Grubb P, Takang AA (2003) Software maintenance: concepts and practice, 2nd edition. Singapore: World Scientific.

[52] Dubois PF (2005) Maintaining correctness in scientific programs. Comput Sci Eng 7: 80–85.

[53] Sanders R, Kelly D (2008) Dealing with risk in scientific software development. IEEE Software 25: 21–28.

[54] List of unit testing frameworks. Available: http://en.wikipedia.org/wiki/List of unit testing frameworks. Accessed September 2013.

[55] Meszaros G (2007) xUnit test patterns: refactoring test code. Boston: Addison-Wesley.

[56] Osherove R (2009) The art of unit testing: with examples in.NET. Greenwich (Connecticut): Manning.

[57] Hook D, Kelly D (2009) Testing for trustworthiness in scientific software. In: Proceedings Second International Workshop on Software Engineering for Computational Science and Engineering. pp. 59–64. 10.1109/SECSE.2009.5069163.

[58] Kelly D, Sanders R (2008) Assessing the quality of scientific software. In: Proceedings First International Workshop on Software Engineering for Computational Science and Engineering. Available: http://secse08.cs.ua.edu/Papers/Kelly.pdf.

[59] Oberkampf WL, Roy CJ (2010) Verification and validation in scientific computing. Cambridge: Cambridge University Press.

[60] Zeller A (2009) Why programs fail: a guide to systematic debugging. Burlington (Massachusetts): Morgan Kaufmann.

[61] Jones MB, Regehr J (1999) The problems you're having may not be the problems you think you're having: results from a latency study of Windows NT. In: Proceedings 7th Workshop on Hot Topics in Operating Systems. pp. 96–101. 10.1109/RTTAS.1999.777681.

[62] Prechelt L (2010) Two comparisons of programming languages. Oram A, Wilson G, editors. Making software: what really works, and why we believe it. Sepastopol (California): O'Reilly. pp. 239–258.

[63] Xie Y (2013). knitr: A general-purpose tool for dynamic report generation in r. R package version 0.9. Available: http://yihui.name/knitr/.

[64] Pérez F, Granger BE (2007) IPython: a system for interactive scientific computing. Comput Sci Eng 9: 21–29.

[65] Fagan ME (1976) Design and code inspections to reduce errors in program development. IBM Syst J 15: 182–211.

[66] Cohen J (2010) Modern code review. Oram A, Wilson G, editors. Making software: what really works, and why we believe it. Sepastopol (California): O'Reilly. pp. 329–336.

[67] Williams L (2010) Pair programming. Oram A, Wilson G, editors. Making software: what really works, and why we believe it. Sepastopol (California): O'Reilly. pp. 311–322.

[68] Dubois P, Johnson J (2003) Issue tracking. Comput Sci Eng 5: 71–77.

[69] Wilson G (2013). Software carpentry: lessons learned. arXiv:1307.5448. Available: http://arxiv.org/abs/1307.5448. 10.12688/f1000research.3-62.v1 PMC3976103 24715981

